# The Ubiquitous Cognitive Assessment Tool for Smartwatches: Design, Implementation, and Evaluation Study

**DOI:** 10.2196/17506

**Published:** 2020-06-01

**Authors:** Pegah Hafiz, Jakob Eyvind Bardram

**Affiliations:** 1 Digital Health Section Department of Health Technology Technical University of Denmark Kongens Lyngby Denmark; 2 Copenhagen Center for Health Technology Kongens Lyngby Denmark

**Keywords:** cognition, memory, response time, attention, Stroop task, wearable devices, mobile phone

## Abstract

**Background:**

Cognitive functioning plays a significant role in individuals’ mental health, since fluctuations in memory, attention, and executive functions influence their daily task performance. Existing digital cognitive assessment tools cannot be administered in the wild and their test sets are not brief enough to capture frequent fluctuations throughout the day. The ubiquitous availability of mobile and wearable devices may allow their incorporation into a suitable platform for real-world cognitive assessment.

**Objective:**

The aims of this study were threefold: (1) to evaluate a smartwatch-based tool for the assessment of cognitive performance, (2) to investigate the usability of this tool, and (3) to understand participants’ perceptions regarding the application of a smartwatch in cognitive assessment.

**Methods:**

We built the Ubiquitous Cognitive Assessment Tool (UbiCAT) on a smartwatch-based platform. UbiCAT implements three cognitive tests—an Arrow test, a Letter test, and a Color test—adapted from the two-choice reaction-time, N-back, and Stroop tests, respectively. These tests were designed together with domain experts. We evaluated the UbiCAT test measures against standard computer-based tests with 21 healthy adults by applying statistical analyses significant at the 95% level. Usability testing for each UbiCAT app was performed using the Mobile App Rating Scale (MARS) questionnaire. The NASA-TLX (Task Load Index) questionnaire was used to measure cognitive workload during the N-back test. Participants rated perceived discomfort of wearing a smartwatch during the tests using a 7-point Likert scale. Upon finishing the experiment, an interview was conducted with each participant. The interviews were transcribed and semantic analysis was performed to group the findings.

**Results:**

Pearson correlation analysis between the total correct responses obtained from the UbiCAT and the computer-based tests revealed a significant strong correlation (*r*=.78, *P*<.001). One-way analysis of variance (ANOVA) showed a significant effect of the N-back difficulty level on the participants' performance measures. The study also demonstrated usability ratings above 4 out of 5 in terms of aesthetics, functionality, and information. Low discomfort (<3 out of 7) was reported by our participants after using the UbiCAT. Seven themes were extracted from the transcripts of the interviews conducted with our participants.

**Conclusions:**

UbiCAT is a smartwatch-based tool that assesses three key cognitive domains. Usability ratings showed that participants were engaged with the UbiCAT tests and did not feel any discomfort. The majority of the participants were interested in using the UbiCAT, although some preferred computer-based tests, which might be due to the widespread use of personal computers. The UbiCAT can be administered in the wild with mentally ill patients to assess their attention, working memory, and executive function.

## Introduction

### Background

Wearable devices provide an opportunity for users to collect their personal data. A recent empirical study determined that fashnology, individuals’ attitudes, and risk context were the most influential factors in adoption of wearable devices for quantified self-tracking purposes [1]. Wrist-worn devices, particularly smartwatches, are becoming more popular. Usefulness and visibility are the two major reasons that people adopt a smartwatch [2]. Smartwatches are lightweight and portable, which makes them easy for people to wear and use in almost every context, while some people may not carry their smartphones when they go for a walk or run. Moreover, platforms, including Fitbit OS (operating system), Apple Watch’s watchOS, and Google's Wear OS, support building stand-alone apps that run without connecting to a smartphone. The application programming interface (API) of some smartwatches allow sensor data collection in the wild, including physiological and behavioral data, such as sleep, heart rate variability, mobility, and location. King and Saffarzadeh reviewed the application of smartwatches in 27 health-related studies [3]. Their findings show that activity monitoring, chronic disease self-management, nursing or home-based care, and health care education are the current smartwatch-based applications in health care. Hence, smartwatches are suitable devices to assist researchers in developing stand-alone health care–related apps, as well as for collecting sensor data in the wild.

Cognitive functioning is a crucial aspect of mental health and determines the quality of individuals’ daily activities. According to Lyon et al, impairment in attention, memory, and executive function may cause problems at school or work [4]. Moreover, previous studies have shown daily fluctuations in alertness [5], working memory [6], and executive skills [7]. Quantifying cognitive performance may help individuals reflect on their own fluctuations. For instance, students can track their alertness levels to select appropriate times of day to schedule their attention-demanding tasks. Besides healthy individuals, mentally ill patients also suffer from cognitive dysfunction, such as dementia [8], bipolar disorder [9,10], attention deficit hyperactivity disorder (ADHD) [11], and schizophrenia [12]. Monitoring cognitive performance can thus help patients in scheduling their follow-up visits in case of significant degradation in their cognitive functioning, as it may indicate the onset of their illness.

Digital cognitive screening tools have been designed for different technological platforms, targeting both mentally ill patients and healthy individuals. The Cambridge Neuropsychological Test Automated Battery (CANTAB) Mobile [13], the Internet-based Cognitive Assessment Tool (ICAT) [14], the THINC-integrated tool (THINC-it) [15], MyCognition Quotient (MyCQ) [16], CogState [17], and the Brief Assessment of Cognition in Schizophrenia (BACS) [18] are some examples of the validated cognitive test batteries administered on a computer or tablet. The existing cognitive test batteries are administered at a certain time in a controlled condition. Such cognitive tools are not feasible for long-term frequent monitoring and assessment of cognitive functioning, since (1) it takes at least 15 minutes to complete a set of tests and (2) the tests are taken in a controlled condition without any distraction, for example, a silent room. However, according to previous studies [19,20], it is crucial to assess cognitive functioning in real-life settings for frequent and continuous monitoring of the individuals.

Ecological momentary assessment (EMA) [21] and the experience-sampling method (ESM) [22] were developed to overcome the bias in delivering retrospective self-reports by study participants. Both methodologies provide an opportunity to collect psychological and clinical measures of behavior, cognition, and emotion in situ [23]. Unobtrusive cognitive tests instead of subjective ratings may improve the accuracy of EMA and the ESM in longitudinal studies.

Taking together, a ubiquitous tool providing continuous and frequent assessment of the individuals' in-the-wild cognitive performance would be an important approach for real-world psychometric research and diagnosis.

### Previous Studies

#### Overview

The application of neuropsychological tests on mobile platforms previously showed promising outcomes [19,24]. In this section, an overview of the previous studies on digital cognitive tests developed for smartphones and smartwatches is presented. Commercial cognitive training mobile apps with no evidence of validity were excluded.

#### Smartphone-Based Tools

A research platform called iVitality includes a smartphone app with five cognitive tests, namely Memory-Word, Trail Making, Stroop, Reaction Time, and N-back. Jongstra et al conducted a study with 151 healthy individuals to examine feasibility and validity of the iVitality platform over 6 months [25]. According to the results of their validation study, the Stroop and Trail Making tests correlated moderately (*r*=.5 and *r*=.4, respectively) with the conventional tests. The authors did not validate the rest of the cognitive tests against their corresponding baseline measures, due to the difference between the raw scores of the smartphone tests and conventional tests. The Color-Shape Test (CST) is a smartphone-based app designed to measure cognitive processing speed and attention in the elderly population. The validity of the CST was examined against the Uniform Data Set (UDS) neuropsychological test battery in an experiment by Brouillette et al with 57 individuals who did not have dementia [26]. Their findings showed a significant correlation between CST scores and global cognition with the Mini-Mental State Examination (*r*=.52), Digit Span (*r*=.43), the Trail Making test (*r*=-.65), and the Digit Symbol test (*r*=.51). However, the CST scores did not correlate with verbal fluency tasks. Tieges et al conducted a study with 20 delirium patients to assess the feasibility of a smartphone-based app called the DelApp against a computerized device called the Edinburgh Delirium Test Box (EDTB) [27]. The authors found no significant difference between the scores of the DelApp and the EDTB (*P*=.41). Pal et al used a mobile app called the Neurophone, which includes N-back, Stop Signal, and Stroop tests, to evaluate the cognitive performance of 20 healthy and 16 methamphetamine users against a validated computerized tool [28]. The Stop Signal test results could not be compared to the computerized tests due to the different parameters used by phone- and computer-based tests, while the scores of the N-back test on both platforms were similar. The authors used speech recognition in the Stroop test of their mobile app to detect the correct response time (RT). However, due to the inaccuracy of the speech recognition, the test results of the computer- and phone-based tests were not comparable. Dingler et al developed a smartphone-based tool including three short cognitive tasks, namely the psychomotor vigilance task (PVT), the go/no-go task, and the multiple object tracking task [29]. The authors conducted an in-the-wild study to assess the alertness of 12 participants over 9 days, on average. Although the short version of the PVT was validated before by Basner et al [30], the go/no-go and the multiple object tracking tasks were not tested against a computer- or paper-based neuropsychological test.

#### Smartwatch-Based Tool

Cormack et al developed a tool called the Cognition Kit for the Apple Watch, including a variation of the N-back test adapted from CANTAB's N-back along with self-reports of mood using a short questionnaire [31]. The authors conducted feasibility and validation studies of the Cognition Kit with 30 depressed patients. According to their validation study results, N-back test performance correlated with CANTAB’s rapid visual information processing task (*P*≤.01, *r*=.5).

### Gaps in the Literature

The study conducted by Dingler et al [29] was the only work that introduced a smartphone-based toolkit for doing research on in situ alertness. The rest of the smartphone-based apps were developed to deliver personal cognitive assessment tools without collecting mobile data. So far, the Cognition Kit is the only smartwatch-based tool exclusively assessing working memory through the N-back test. A limited number of cognitive measures provided by mobile tools, as well as a lack of studies in exploring the potential of smartwatches in measuring in-the-wild cognition, led us to build the Ubiquitous Cognitive Assessment Tool (UbiCAT). Our tool has three smartwatch-based cognitive tests measuring three key cognitive domains, namely attention, working memory, and executive function. The UbiCAT tests, along with smartwatch-based sensor data collection, allow researchers to analyze associations between individuals’ cognitive, physiological, and behavioral features toward identifying digital biomarkers of human cognitive functioning and conducting psychometric research in the wild.

### Goals of This Study

Through this study, we will (1) evaluate the cognitive measures of the UbiCAT apps against state-of-the-art computer-based tools, (2) assess the usability of the UbiCAT tests, and (3) understand participants' perceptions about smartwatch apps for assessing cognition.

## Methods

In this section, we first provide details of the design and functionality of the UbiCAT apps; we then explain the study in detail.

### Design Methods

#### Overview

The UbiCAT includes three smartwatch-based apps; each is a cognitive test that measures a certain cognitive domain. We considered three inclusion criteria for the UbiCAT tests: (1) the tests should measure memory, attention, and executive function, since fluctuations in these domains may negatively affect individuals’ work or study performance, (2) each test should be able to be adapted for the limited screen size of the smartwatch, and (3) each test should not require a microphone or speaker, which are essential in verbal recall tests. Taking these together, we selected a two-choice reaction-time test [32] to measure attention, the Stroop color-word test [33] to measure attention and executive function, and the N-back test [34] to examine working memory. The three tests contribute to short assessments, as it takes approximately 5 minutes to take the UbiCAT tests.

Three experts who each hold a doctoral degree within cognitive psychology and human-computer interaction were involved in the design process. First, the initial design of the aforementioned tests was sketched on paper. Based on detailed analysis of the available smartwatch hardware platforms, the Fitbit Ionic device was selected. Second, functional prototypes for each test were implemented separately and tested on the smartwatch. Individuals with different finger sizes were asked to work with the apps to adjust the size of the app buttons and text. The Fitbit design guidelines were also considered during the prototyping phase. The components of the UbiCAT apps were revised several times after meetings with the domain experts. Overall, the design and implementation process took 4 months. Third, a formative evaluation study of the earlier versions of the UbiCAT apps was conducted with 5 participants aimed to examine the usability of the apps and understand participants' adoption of wrist-worn devices [35]. The findings of the formative evaluation study helped us improve the user interface and functionality of the apps.

#### The UbiCAT Cognitive Tests

##### Overview

**Figure 1:**
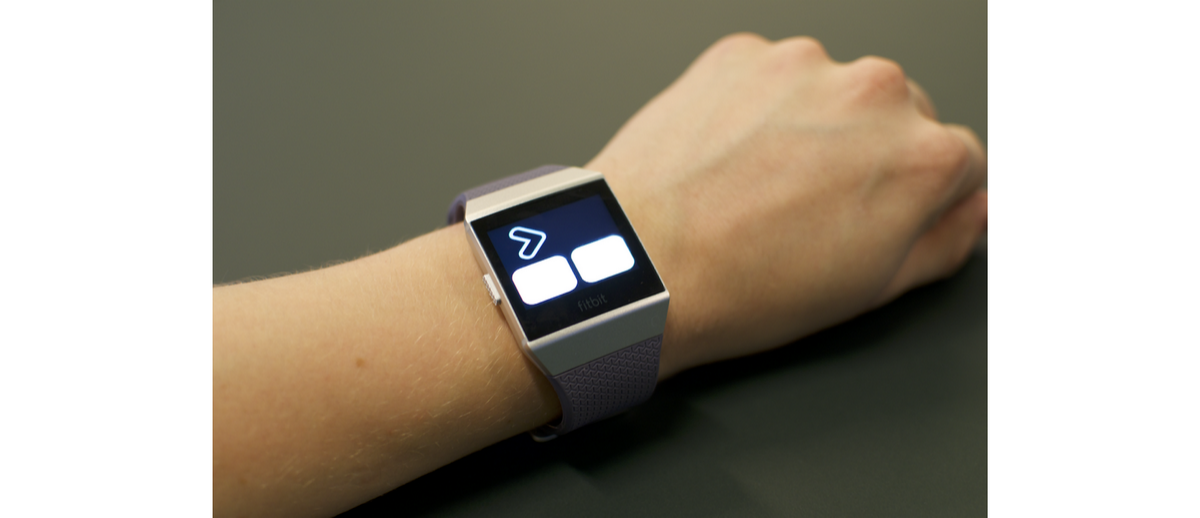
A sample test taken from the UbiCAT (Ubiquitous Cognitive Assessment Tool) Arrow test. The stimuli is the rightward arrow and the app buttons on both sides capture the direction of the arrow.

**Figure 2:**
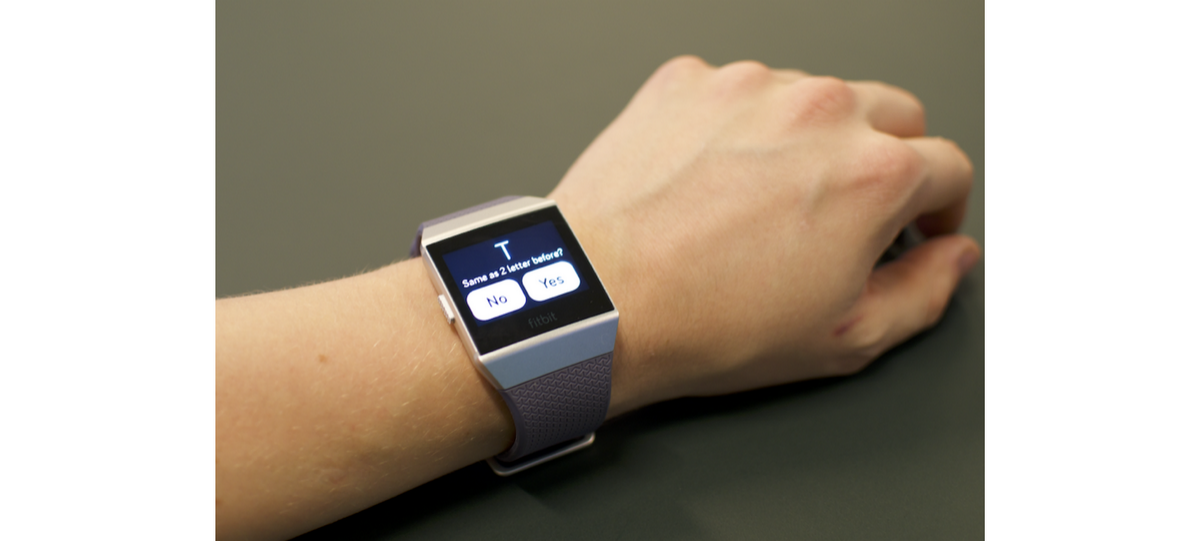
A sample test taken from the UbiCAT (Ubiquitous Cognitive Assessment Tool) Letter test, 2-back task. The participant should indicate whether “T” appeared 2 letters back in the sequence or not.

**Figure 3:**
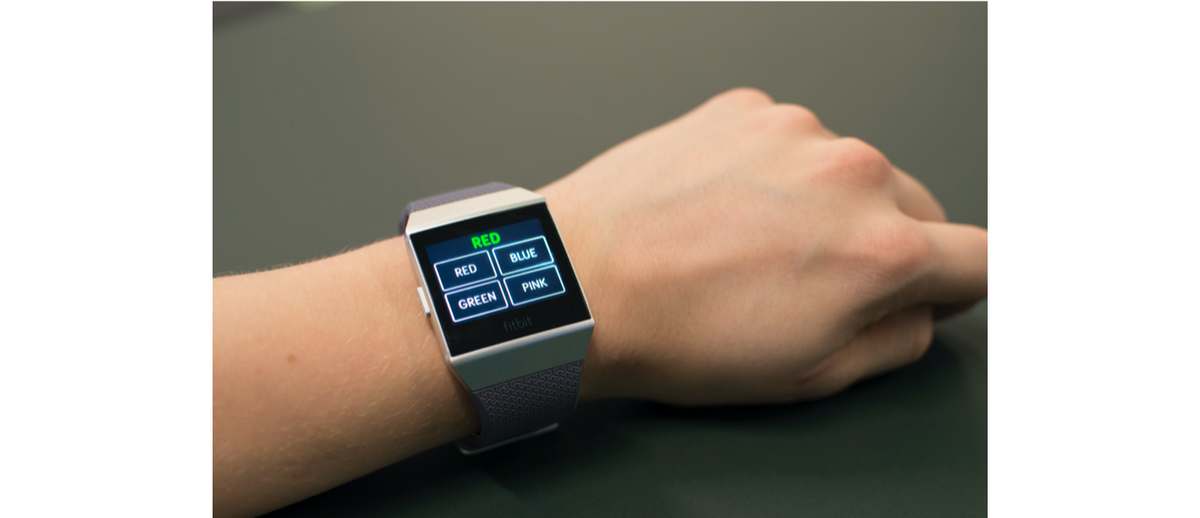
A sample test taken from the UbiCAT (Ubiquitous Cognitive Assessment Tool) Color test, displaying an incongruent stimuli.

Three stand-alone apps were built for the Fitbit smartwatch. Each test takes less than 2 minutes to complete. We selected the following names for the UbiCAT apps to simplify memorizing the apps for the users: *Arrow test* (two-choice reaction-time test), *Letter test* (N-back test), and *Color test* (Stroop color-word test). An outline of the UbiCAT apps is presented in the following sections and snapshots are shown in Figures 1-3.

##### Arrow Test

The Arrow test presents a sequence of rightward or leftward arrows to the user one by one. The user is required to select the correct direction of each arrow by tapping on either the left or right app button. The position of each arrow can be on the left or right side of the screen. Figure 1 shows a snapshot of the Arrow test where the correct response to this stimulus is the app button on the right side.

##### Letter Test

In the Letter test, a sequence of English alphabet letters are displayed to the user. Depending on the value of N, the user is supposed to determine whether the current stimulus is the same as the N letter, or N letters, back in the sequence or not. The value of N determines the difficulty level and is unchanged during an entire trial. Figure 2 shows a snapshot of the 2-back test, where N is equal to 2.

##### Color Test

The names of four colors, for example *RED*, with either the same or different ink color are the stimuli of the Color test. A congruent stimulus has the same color as its meaning, while an incongruent stimulus has a different color. The task of the user is to select the ink color of each stimulus by tapping on the app button labeled with the color name. Figure 3 presents an incongruent stimulus. Here, the correct response is the *GREEN* app button in the bottom-left corner.

#### Technical Specifications and Apparatus

Two validated computer-based tools, PsyToolkit [36,37] and the THINC-it application [15], were run on a MacBook Pro (15-inch Retina display, Apple Inc) during the study. A Fitbit Ionic smartwatch (1.42-inch screen, 348 × 250 pixel resolution) was used to run the UbiCAT apps. The figures in this paper were created in RStudio using the ggplot2 package [38].

#### Ethical Approval

The study protocol and system description were sent for approval by the Danish Ethical Committee. The study was classified as a nonclinical survey study and, hence, exempted for ethical approval (Journal-nr.: H-19086232).

### Participant Recruitment

We recruited 21 healthy adults who lived in Copenhagen, Denmark, using a snowball sampling method [39]. All participants had sufficient English-language skills to read the test instructions. Participants were not eligible if they had a history of mental illness, were aged over 50 years, or had color blindness.

### Procedure

#### Overview

**Figure 4:**
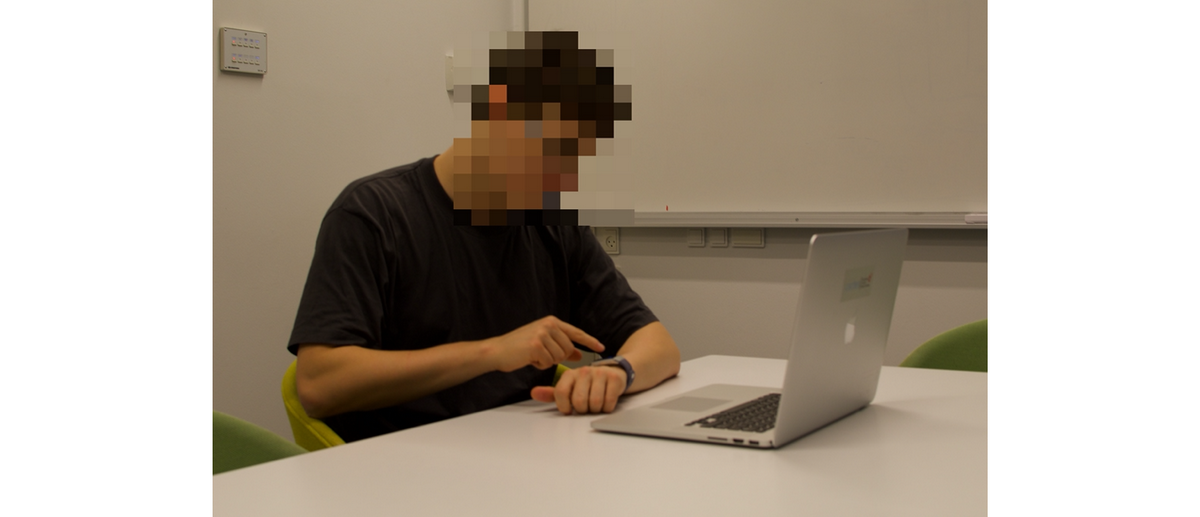
A study participant completing a UbiCAT (Ubiquitous Cognitive Assessment Tool) test via a Fitbit Ionic smartwatch. The laptop was used to administer computer-based tests.

All of the test sessions were performed in a silent room at the Technical University of Denmark. The study session lasted 60-75 minutes per participant. Participants were compensated with a gift card worth an amount equal to US $15 that was given at the end of the study. Prior to an experiment, the study leader (PH) informed the participant to ask for a short break between the testing sessions if needed. We measured each participant’s perceived wrist discomfort after completing each of the UbiCAT tests using a 7-point Likert scale. Figure 4 shows a participant completing a UbiCAT test on the Fitbit smartwatch. A detailed description of the experiment is presented below.

First, a general description of the study was given to the participant. Second, a consent form was handed to the participant. Upon signing the consent form, background information from the participant was collected, including age, gender, educational background, and preference in terms of watch-wearing wrist (ie, dominant or nondominant hand). Third, the participant was asked to perform the three UbiCAT tests one by one. Each test was administered against its corresponding computer-based test. PH explained the instructions of the computer-based tests to the participant and repeated if needed. The participant was able to read the instructions of the smartwatch-based tests in the UbiCAT by themself. The feedback displayed to the participant was the fraction of correct responses to the total responses in each UbiCAT test. The interaction of each participant with the smartwatch was video-recorded during the experiment. The order of test administration on the smartwatch and computer was counterbalanced between participants.

Previous work mentioned that some of the cognitive test results obtained from paper-based and computer-based tools could not be compared to their corresponding smartphone-based tests, due to the difference between parameters. Therefore, we chose the PsyToolkit for the Stroop color-word test and the N-back test. This tool allows researchers to program their experiments and adapt the parameters to their needs. We matched the difficulty levels of the N-back tests by changing the N and selecting the same ratio of congruent stimuli to the incongruent stimuli (1:3) in the Stroop tests. The details of our study are presented below.

#### Arrow Test Versus Spotter Test

All participants took the Arrow test and THINC-it Spotter test twice. The stimuli of each test on both the smartwatch and the computer was a set of 40 arrows. Each arrow was displayed on the watch for a maximum of 2000 ms. The interstimulus interval was randomly selected to be between 1000 and 3000 ms. The input of the THINC-it Spotter test was received by pressing the left or right arrow key, while the input was captured by tapping the left or right app button in the UbiCAT Arrow test. The performance measure calculated for both tests was the number of correct responses and fastest RTs.

#### Letter Test Versus PsyToolkit N-Back Test

The N-back test was administered separately with three difficulty levels, starting from N=1. The tests with the same difficulty level were tested against each other. For instance, 1-back in the Letter test was examined against the PsyToolkit 1-back test. The stimuli of each test was a sequence of 40 English alphabet letters displayed one by one. The time limit for the participant to respond to a stimulus was 2500 ms. Two keys were used to respond during the PsyToolkit test: “m” for yes and “n” for no. The inputs were captured on the UbiCAT Letter test by tapping on the app buttons labeled as “Yes” and “No.” The performance measures were the number of correct responses and mean RTs to the stimuli.

#### Color Test Versus PsyToolkit Stroop Test

All participants took each test twice. The stimulus of each test was 30 color names consisting of 7 congruent and 23 incongruent color names. The time limit was 2500 ms. Participants were required to press “b” for blue, “g” for green, “r” for red, and “y” for yellow in the PsyToolkit Stroop test. Responses were captured in the UbiCAT Color test by tapping on the app buttons labeled with the color names (see Figure 3). The pink color replaced yellow on the Fitbit smartwatch for some participants who found yellow difficult to distinguish. The performance measures of the Stroop tests were the mean RTs to the congruent and incongruent stimuli.

#### Usability Testing

The usability of the UbiCAT apps was assessed using the Mobile App Rating Scale (MARS) questionnaire [40]. Relevant questions concerning aesthetics, functionality, and information were selected from the MARS questionnaire (see Multimedia Appendix 1). The rating scale for each of the MARS questions ranged from 1 (the lowest score) to 5 (the highest score).

#### Perceived Cognitive Workload

Each N-back task was preceded by the NASA-TLX (Task Load Index) questionnaire [41] to quantify participants' perceived cognitive workloads using a 7-point Likert scale. The following subscales of the NASA-TLX were used: mental demand, temporal demand, performance, effort, and frustration level. It should be noted that the physical demand subscale was excluded as it was deemed irrelevant.

#### Follow-Up Interview

Upon finishing each experiment, a short interview was performed with each participant to investigate their subjective perception about the experiment and the UbiCAT tests, as well as their suggestions to improve the apps and/or instructions. The interviews were audio-recorded and transcribed for semantic analysis and grouping of the findings across participants.

### Statistical Analysis

The Pearson correlation test was performed on the number of correct responses and mean RTs of the cognitive tests on both platforms. The paired-sample *t* test was applied on the performance measures to compare the numbers obtained from the smartwatch- and computer-based tests. One-way analysis of variance (ANOVA) was used to analyze the effect of difficulty level on the participants' test performances during the N-back test. The CI of the statistical tests was 95%. The statistical analysis was performed in JASP, version 0.11.1 (The JASP Team).

## Results

### Participant Statistics

Participants were aged between 19 and 44 years (mean 26, SD 6), and 9 out of 21 participants (43%) were female. On average, participants spent 5.7 years studying at a higher-education level. Participants had diverse occupational backgrounds, including design, computer science, water engineering, construction, health care, energy, and food engineering. Of the 21 participants, 10 (48%) of them had used at least one wrist-worn device before. All participants except for 1 (20/21, 95%) wore the smartwatch on their nondominant hand.

### Overall Analysis

**Figure 5:**
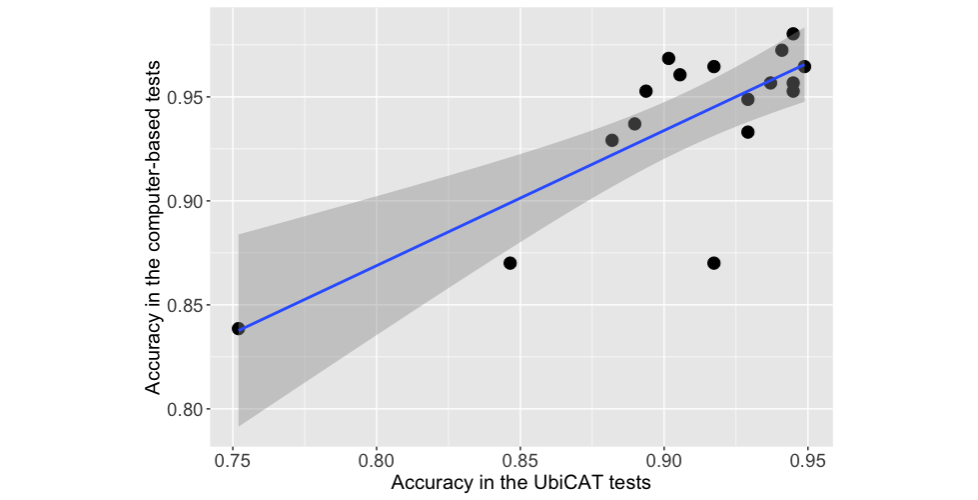
Overall participant accuracy in the three cognitive tests. Each black dot represents results from one participant. The blue line is the regression line and the shaded region is the CI. UbiCAT: Ubiquitous Cognitive Assessment Tool.

Pearson correlation analysis revealed a significant strong correlation between the total number of correct responses obtained from the cognitive tests on the UbiCAT and computer-based tools (*r*=.78, *P*<.001). It should be noted that the scores of 4 participants of the PsyToolkit Stroop test were lost; thus, the correlation analysis between the total scores was performed for 17 participants. Figure 5 shows the total participant accuracy obtained from the UbiCAT apps versus the computer-based tools, along with the regression line. The single data point located on the bottom-left corner of Figure 5 might indicate an outlier; however, we did not remove this sample point, since it is normal that the abilities of the individuals are different from each other.

### Two-Choice Reaction-Time Tests

**Figure 6:**
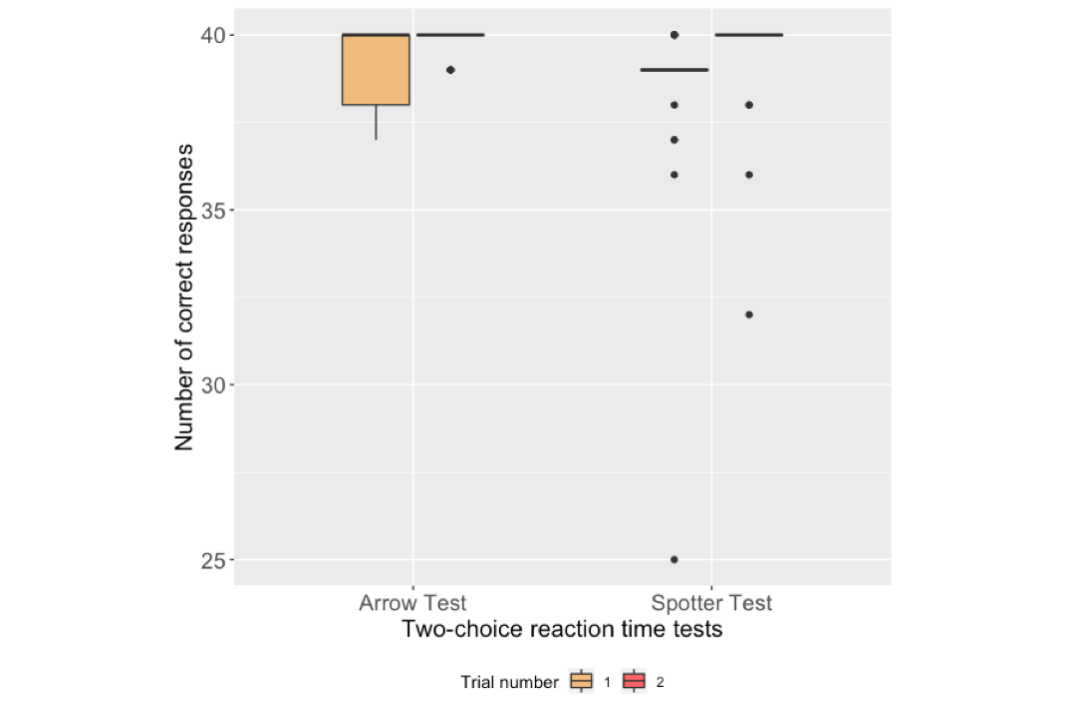
Box plots of participants’ number of correct responses during the two-choice reaction-time tests.

**Figure 7:**
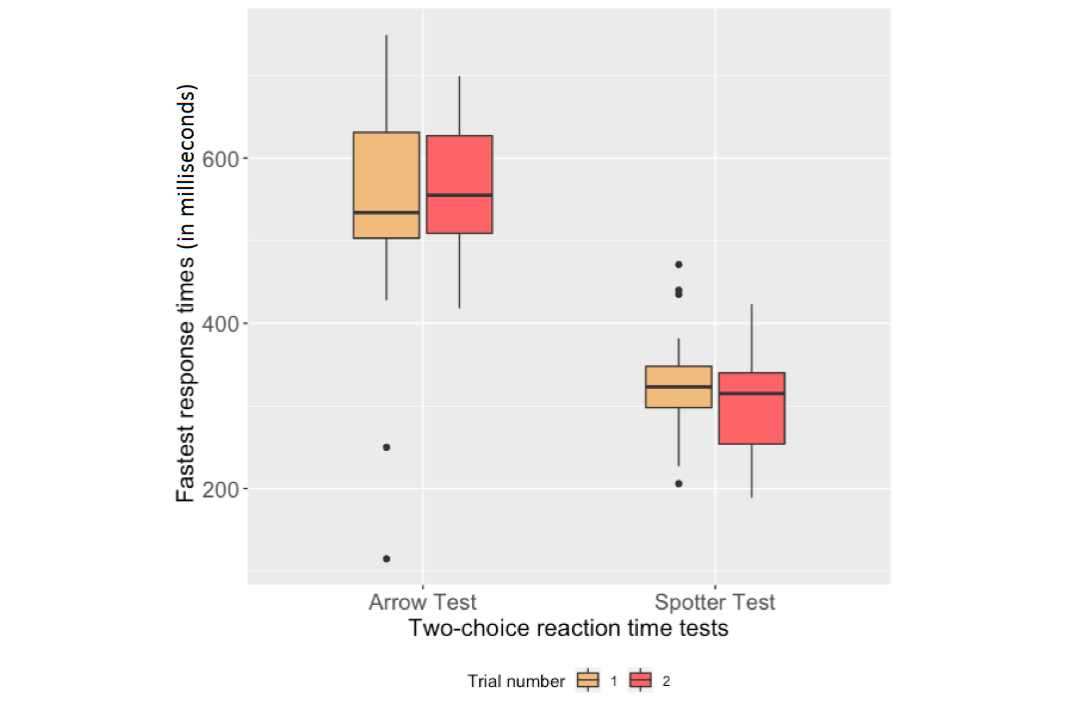
Box plots of participants’ fastest response times during the two-choice reaction-time tests.

The Pearson correlation analysis that was applied on the average of correct responses in the two trials of the Arrow test and Spotter test and the participants' fastest RTs on both platforms is presented in Table 1. Figure 6 shows the box plots of the number of correct responses for both platforms during each trial. Figure 7 shows the box plots of the participants' fastest RTs calculated for both trials of the two-choice reaction-time tests. We applied the paired-sample Student *t* test and it revealed that the fastest RTs obtained from the Arrow test in both trials were not statistically different (t_20_=-1.266, *P*=.22). The average of the participants' fastest RTs in the Arrow test were statistically higher than in the Spotter test (t_20_=10.84, *P*<.001).

### N-Back Test

**Figure 8:**
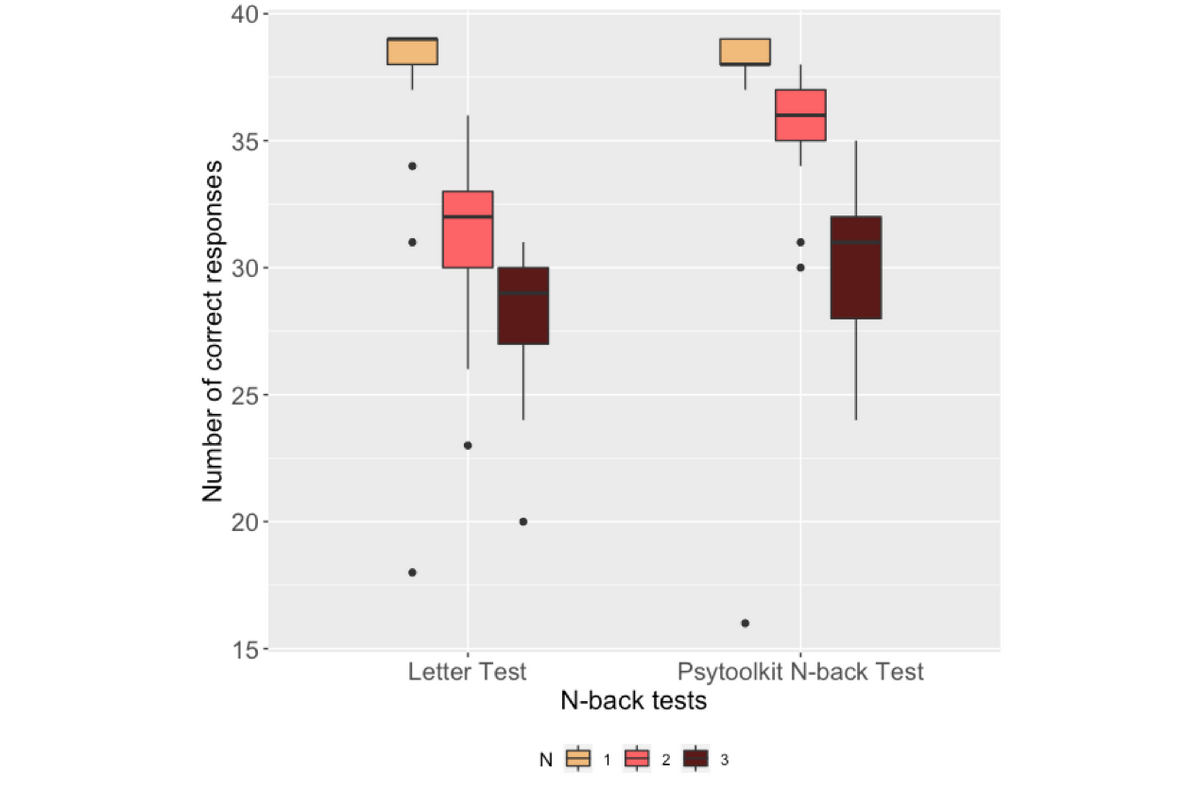
Box plots of participants' number of correct responses in the N-back tests.

**Figure 9:**
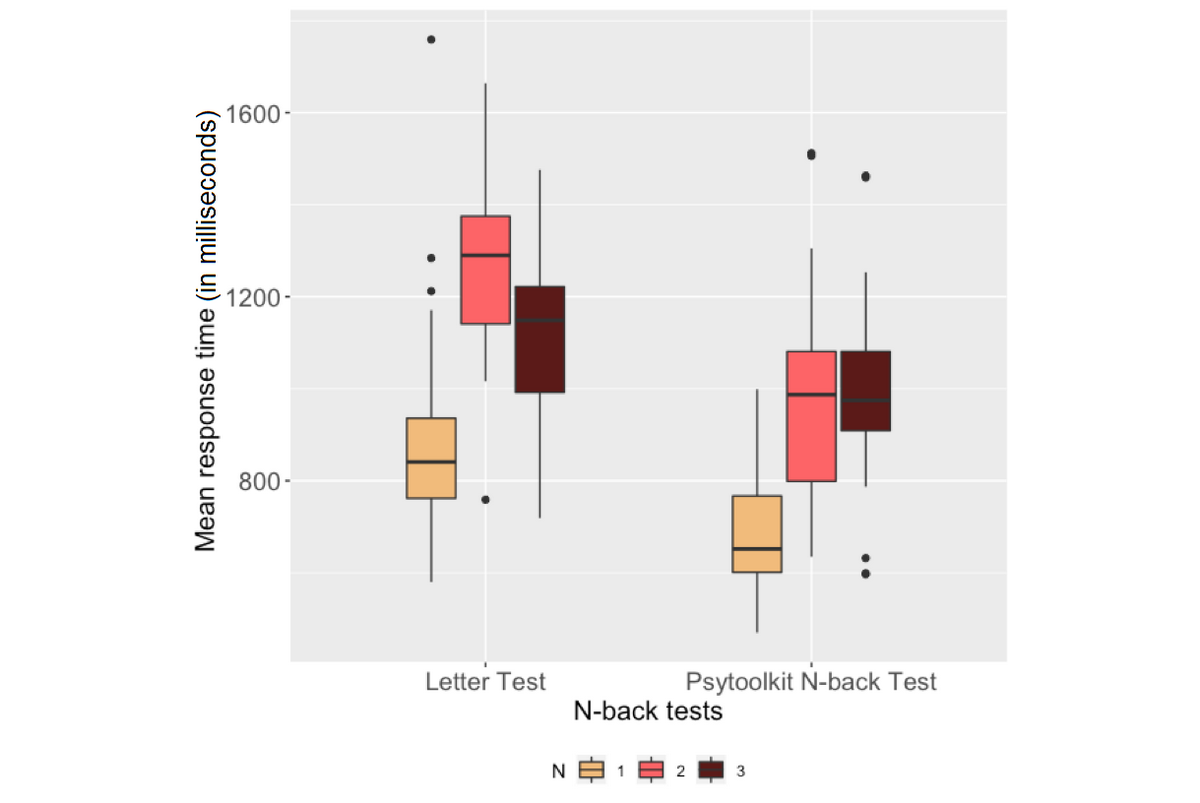
Box plots of participants' mean response times during the N-back tests.

Figures 8 and 9 show the number of correct responses and the mean RTs of the participants, respectively, during the 1-back, 2-back, and 3-back tests in the Letter test and the PsyToolkit N-back test. Pearson correlation analysis was performed on the number of correct responses and the mean RTs for each difficulty level between the Letter test and PsyToolkit N-back test. The results are presented in Table 2.

**Table 1:** Correlation analysis between performance measures in the Arrow test and the Spotter test.

Performance measure	Pearson *r*	*P* value
Average of correct responses	.61	.003
Fastest response times	.24	.30

**Table 2:** Correlation analysis between performance measures of the N-back tasks in the Letter test and PsyToolkit N-back test.

Performance measure and tasks		Pearson *r*	*P* value
**Mean response time**			
	1-back	.78	<.001
	2-back	.71	<.001
	3-back	.53	.01
**Number of correct responses**			
	1-back	.90	<.001
	2-back	.19	.40
	3-back	.35	.13

One-way ANOVA was performed to analyze the effect of difficulty level on the participants' test performances (see Multimedia Appendix 2).

The results of the NASA-TLX questionnaire for 1-back, 2-back, and 3-back on both platforms are reported in Table 3. The numbers in this table show the means and SDs calculated based on the 7-point Likert scales for the metrics of the NASA-TLX.

**Table 3:** The N-back cognitive workload results using the NASA-TLX (Task Load Index) metrics.

Device and task		Score^a^ for each metric, mean (SD)				
		Mental demand	Temporal demand	Overall performance	Effort	Frustration level
**Smartwatch**						
	1-back	2.81 (1.50)	2.81 (1.29)	2.05 (1.32)	3.05 (1.20)	2.52 (1.60)
	2-back	4.71 (1.27)	4.19 (1.50)	4.29 (1.49)	4.43 (1.17)	3.86 (1.80)
	3-back	5.19 (1.33)	4.05 (1.75)	4.67 (1.62)	5.10 (1.10)	3.95 (1.80)
**Computer**						
	1-back	2.76 (0.99)	2.86 (1.62)	2.91 (1.84)	2.67 (1.07)	2.48 (1.47)
	2-back	4.50 (1.54)	3.50 (1.61)	3.10 (1.52)	4.35 (1.35)	2.95 (1.64)
	3-back	5.52 (1.29)	4.24 (1.76)	4.76 (1.76)	5.00 (1.18)	4.00 (1.73)

^a^Scores were based on the 7-point Likert scales of the NASA-TLX metrics.

### Stroop Color-Word Test

**Figure 10:**
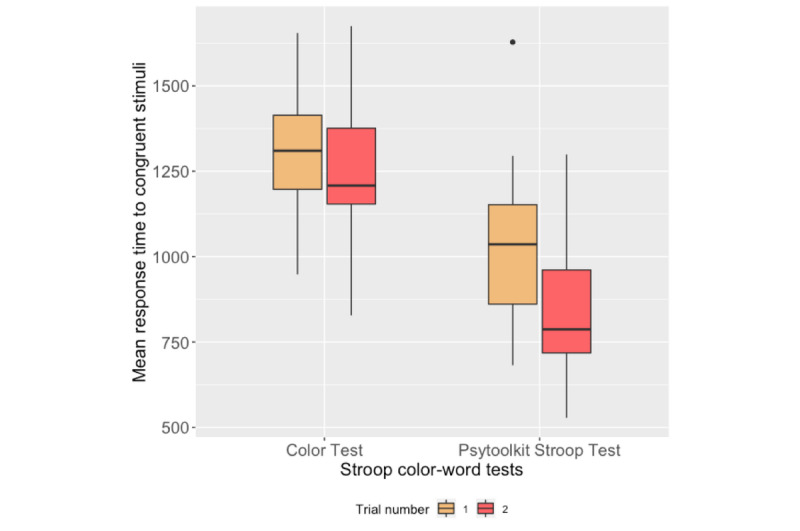
Box plots of participants' mean response times to congruent stimuli during Stroop tests.

**Figure 11:**
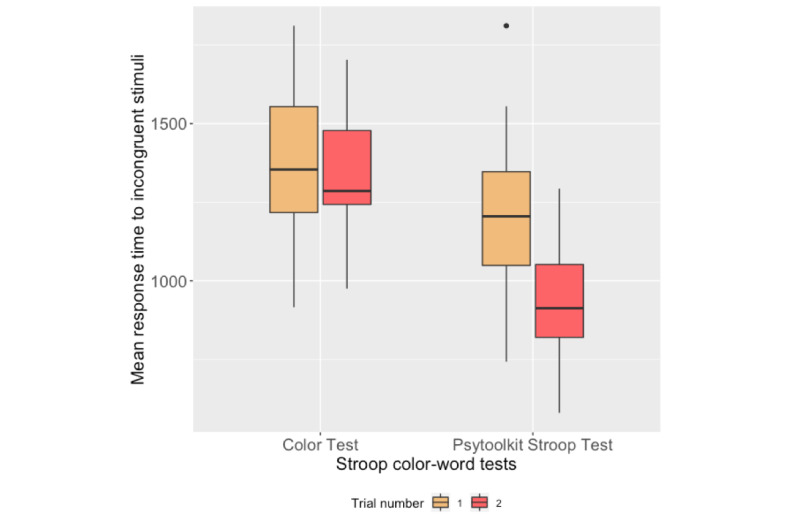
Box plots of participants’ mean response times to incongruent stimuli during the Stroop tests.

**Figure 12:**
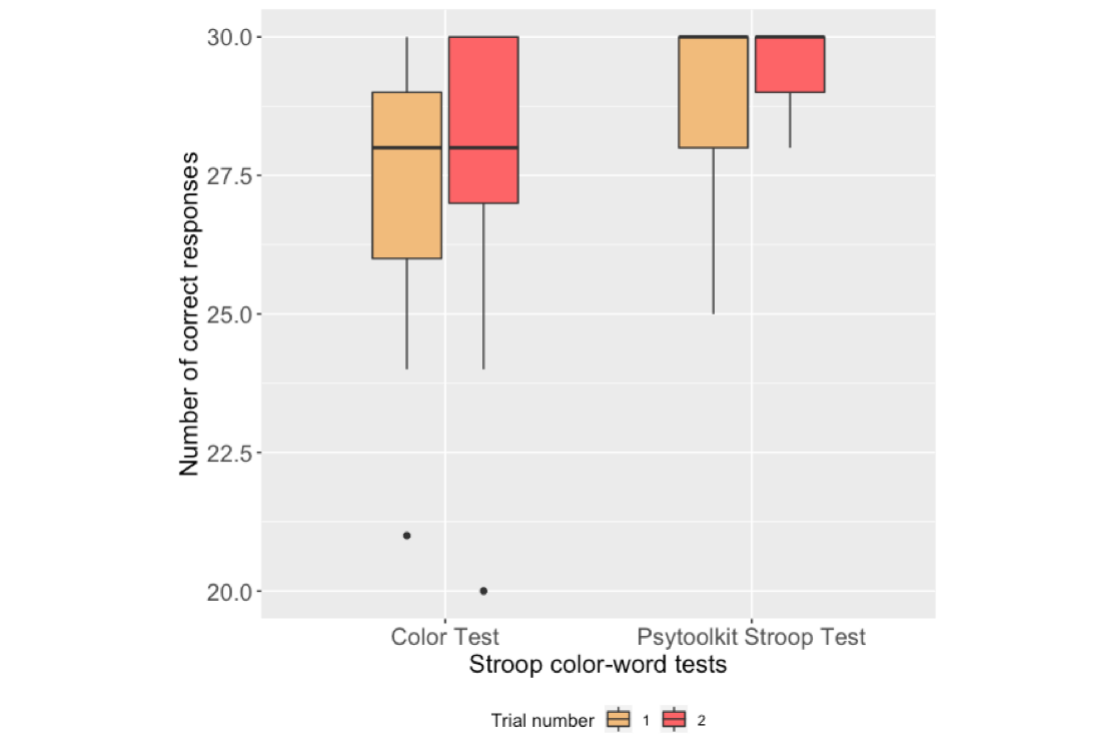
Box plots of participants’ number of correct responses in the Stroop tests.

Figures 10 and 11 present the box plots of the mean RTs to the congruent and incongruent stimuli for each trial of the Color test and the PsyToolkit Stroop test, respectively. Table 4 reports the correlation analysis between the performance measures of the Stroop tests on both platforms. Box plots of the number of correct responses to both congruent and incongruent stimuli are shown in Figure 12.

### Usability Ratings

The psychometric factors considered for the usability test were aesthetics, functionality, and information. Each of the UbiCAT apps were rated separately by the participants. Table 5 reports the means and SDs of the usability ratings, which are out of 5.

**Table 4:** Correlation analysis between performance measures in the Color test and the Stroop color-word test.

Performance measure	Pearson *r*	*P* value
Mean response times to congruent stimuli	.67	<.001
Mean response times to incongruent stimuli	.66	.001

**Table 5:** Usability ratings of the UbiCAT (Ubiquitous Cognitive Assessment Tool) apps.

UbiCAT app	Score^a^ for each factor, mean (SD)		
	Aesthetics	Functionality	Information
Arrow test	4.02 (0.76)	4.55 (0.52)	4.24 (0.86)
Letter test	4.19 (0.75)	4.36 (0.62)	4.33 (0.60)
Color test	4.14 (0.83)	4.64 (0.45)	4.31 (0.64)

^a^Scores ranged from 1 (the lowest score) to 5 (the highest score).

### Perceived Discomfort

For each UbiCAT app, participants rated the discomfort level in their wrist on which they wore the smartwatch via a 7-point Likert scale from 1 (the least discomfort) to 7 (the most discomfort). The corresponding means and SDs of the discomfort levels, calculated separately for the Arrow test, Letter test, and Color test, are 2.71 (SD 1.79), 2.24 (SD 1.18), and 2.14 (SD 1.32), respectively.

### Interviews

#### Overview

Seven themes were extracted from the participants' responses and a brief description of each theme is presented below. The participants’ quotes are presented in Multimedia Appendix 3.

#### Perceptions About the Experiment

Participants were asked to describe their feelings about the experiment. They were generally engaged in the experiment: 5 participants out of 21 (24%) mentioned that the experiment was “fun,” 3 (14%) said it was “good,” and 3 (14%) said it was “fine.” Only 1 participant out of 21 (5%) believed that the experiment was too long. The rest of the participants did not express their opinions or had to leave immediately after the experiment.

#### Input Modality

Participants compared the input modalities of the smartwatch and computer. Participants #1 and #3 (2/21, 10%) preferred the app buttons of the UbiCAT Color test to the keyboard in the Stroop test. Participants #9, #12, and #19 (3/21, 14%) felt more comfortable with the app buttons in general, and participant #14 (1/21, 5%) liked the tangibility of the keyboard.

#### Device Screen

Some participants compared the screen size of the smartwatch with the computer. Out of 21 participants, 1 (5%) argued that the bigger screen of the computer influenced his or her performance positively and 2 (10%) preferred the screen size of the computer to the smartwatch. We understood that computer screen size might be more acceptable for some people due to the longer adoption time of personal computers compared to smartwatches.

#### Visual Impact

Out of 21 participants, 3 (15%) implied that their better performance on the computer was due to the visualization of the elements. A participant (1/21, 5%) did not like the visual elements of the Fitbit, indicating that the overall device design and graphics affected participants' interaction quality apart from the specific user-interface design of the UbiCAT apps.

#### Psychological Factors

Apart from the physical characteristics of a smartwatch and a computer, psychological factors also influenced participants' performance. A participant (1/21, 5%) pointed to the gamified nature and playfulness of the UbiCAT tests.

#### Performance

Some of the participants related their lower performance in the UbiCAT tests to the apps. Out of 21 participants, 4 (19%) mentioned that the Color test sometimes did not capture their taps on the app buttons during the test. We noticed such an incident while reviewing the records of the experiments. The position of the app buttons in the Color test changed randomly to avoid practicing the positions of the buttons. It surprised some of the participants during the test. Besides, 1 of the participants (5%) thought that his or her performance might differ significantly between the first and second trials of the cognitive tests.

#### Suggestions

Of the 21 participants, 3 of them (14%) proposed suggestions regarding the font size used in the UbiCAT tests.

## Discussion

### Principal Findings

UbiCAT implements three smartwatch-based cognitive assessment tests for in-the-wild deployment. The findings of this study revealed comparable performance measures to computer-based tests. The strong correlation between the overall accuracy of the participants during the cognitive tests in the UbiCAT and computerized tools showed that UbiCAT can be utilized for assessing individuals' three key cognitive functions, namely attention, working memory, and executive function. The analysis between the following performance measures of the UbiCAT and computerized tests revealed significant correlation coefficients: the number of correct responses in the two-choice reaction-time test; mean RTs in the 1-back, 2-back, and 3-back tests; the number of correct responses in the 1-back test; and the mean RTs to the Stroop test’s congruent and incongruent stimuli.

The psychometric factors, including aesthetics, functionality, and information quality and quantity, of the UbiCAT apps had high average ratings by the participants (>4 out of 5). The subjective ratings of the participants' wrist discomfort levels were less than 3 out of 7, indicating that interaction with the UbiCAT apps via the smartwatch was comfortable, which is in line with our overall objective of making cognitive assessment as simple and convenient as possible.

Previous work reported mobile cognitive test results along with paper-based or computerized tests. Comparison between the correlation coefficients reported in previous studies and in our study is not possible due to different parameters, number of participants, and target population. Nevertheless, our test outcomes obtained from computer- and smartwatch-based apps were comparable to each other, unlike some of the previous studies (eg, Neurophone Stop Signal test) that could not compare their results with computerized or paper-based tests due to dissimilar parameters.

### Two-Choice Reaction-Time Test Outcomes

The average number of correct responses obtained from the THINC-it and Arrow tests correlated significantly with each other. As it can be seen in Figure 6, the majority of the participants received the highest score on both platforms, which may indicate a ceiling effect. The participants' fastest RTs, however, did not correlate with each other, which might be due to the different interaction methods on both platforms. The app buttons in the Arrow test (see Figure 1) disappeared on receiving an input or time-out until the next stimulus appeared, since an accidental tap on the buttons could impede calculating the real performance of the participants. We observed that the participants moved their index fingers away after tapping on an app button in the Arrow test, while they kept their fingers on the arrow keys on the computer keyboard during the THINC-it Spotter test. Such a difference between the users' interactions may explain the longer RTs of the UbiCAT Arrow test as compared to the THINC-it Spotter test. Nevertheless, the difference between the fastest RTs of the participants helped us in understanding the impact of interaction methods.

The fastest RTs measured via both platforms may indicate that the thresholds of individuals' alertness vary on the computer and smartwatch platforms. In our study, the average fastest RTs of the participants in the Arrow test was 545 ms (SD 88), while the corresponding result for the THINC-it Spotter test was 315 ms (SD 59). A study on the development of a brief version of the PVT (PVT-B) showed that 500 ms might be the threshold for an impaired alertness [30], which is in line with the average fastest RTs obtained from the THINC-it computer-based test. However, the participants of the PVT-B study pressed a button to respond during the tests, which is similar to the interaction method of the THINC-it Spotter test. Therefore, this threshold may not be comparable to the fastest RTs obtained from the Arrow test on the smartwatch. To infer the level of impairment on the basis of the user's fastest RT delivered via a smartwatch, a larger study is required that would include both healthy controls and cognitively impaired patients. Nevertheless, the findings of our study revealed that the average fastest RT of the healthy subjects to a smartwatch-based test is above 500 ms.

According to Figure 7, participants’ fastest RTs were almost the same during the first and second trials of the Spotter test (327 ms and 303 ms, respectively), while their responses were a bit slower in the second trial of the Arrow test (564 ms) compared to the first trial (526 ms). A paired-sample *t* test showed that the fastest RTs received from the Arrow test in both trials were not statistically different.

### N-Back Test Outcomes

One-way ANOVA showed the effect of difficulty level on the number of correct responses and mean RTs in the UbiCAT Letter test and the PsyToolkit N-back test. The perceived cognitive workload in the N-back tests also revealed that as N-back tasks became more difficult, the participants' cognitive workload increased. The mean RTs obtained from each N-back task on both the smartwatch and the computer correlated significantly. Figure 9 shows that the mean RT during the UbiCAT 2-back test was higher than that of the 3-back test, while statistical analysis revealed no significant difference between the mean RTs of the UbiCAT 2-back and 3-back tests. The RTs of the PsyToolkit 2-back and 3-back tests were not statistically different either (*P*>.99). According to Table 3, higher temporal effort reported through NASA TLX questionnaires for the 2-back Letter test compared to the 3-back test may imply that participants were more rushed during the 2-back test. Moreover, participants might have spent more time on practicing the 2-back test right after taking the 1-back test to adapt their mental skills, since the reported mental effort for both 2-back and 3-back tests were higher than for the 1-back test on both the computer and the smartwatch.

According to Table 2, the correlation analysis between the number of correct responses of the N-back tests on the smartwatch and the computer was only significant for the 1-back test. The lack of a significant correlation between the 2-back and 3-back tasks might be due to the N-back test itself, since the letter sequences of the N-back test were generated randomly and the maximum number of matches (ie, hits) during the N-back tests was not controlled to be the same between the computer and the smartwatch.

### Stroop Test Outcomes

The RTs to the congruent and incongruent stimuli on the PsyToolkit and Color tests significantly correlated with each other. According to Figures 10 and 11, the RTs obtained from the second trials were lower than the first trials for both the PsyToolkit Stroop test and Color test. However, the magnitude of difference between the RTs in both trials of the Color test was lower than that in the PsyToolkit Stroop test. It might be due to the difference between the interaction methods of the tests. In the Color test, the order of app buttons was shuffled after a test run to avoid practicing the positions and increasing engagement with the apps. On the other hand, the position of the keys was obviously stable during the PsyToolkit tests. Hence, participants might get used to the position of the keys and respond faster in the second trial of the PsyToolkit Stroop test, while the changing position of the app buttons in the Color test took some time for them to practice with the new positions. The change in the position of the app buttons was intended to obtain reliable outcomes during future studies for longitudinal frequent administration.

Figure 12 shows that several participants received the highest score in the PsyToolkit Stroop tests (ie, 10 participants in trial 1 and 11 participants in trial 2), while the scores are more distributed in the Color tests (ie, 2 participants received the highest score in trial 1 and 6 participants in trial 2). In addition, we observed that sometimes the app buttons in the Color test did not capture touch inputs by the participants and some participants reported this issue during the interviews. Therefore, lower scores in the Color test might be due to the Fitbit’s touch sensitivity.

### Perceptions From the Interviews

Seven themes were identified from the follow-up interviews with the participants. Some of the participants generally felt more comfortable when taking a cognitive test on the smartwatch compared to the computer, while some did not. Factors related to the physical aspects of the device, including the screen size and distance and the input modalities, affected their interactions. We understood that longer adoption times of computers compared to smartwatches may explain why some participants preferred computer tests to the UbiCAT apps. Therefore, deploying smartwatches into individuals’ daily lives may take some time and may not be useful for all. Psychological factors were also involved in determining participants’ engagement with UbiCAT, such as the gamified features of the tests.

### Implications for Future Work

On the basis of our interviews, we decided to (1) add customized badges to the UbiCAT apps depending on participants’ test performances to motivate them toward continuous usage of the UbiCAT, (2) increase the font size of the stimulus in the Letter test since it was not easy for some of the participants to read, and (3) keep the right and left app buttons of the Arrow test on the screen after they tap on a button.

This study was conducted to evaluate our novel smartwatch apps against their corresponding computer tests, as well as to investigate participants' perceptions about the study and usability of the UbiCAT apps. One of the future directions of the UbiCAT project is to identify digital biomarkers of human cognition. In an upcoming study, we will collect participants’ mobile data, including physiological and behavioral data, along with assessing their daily cognitive functioning through the UbiCAT apps to determine digital biomarkers of human cognition. The digital biomarkers would help researchers in building predictive models of individuals' cognitive impairment using mobile data.

Sleep-stage data and heart rate variability (HRV) are the physiological data that can be collected through the Fitbit API. Sleep disturbance is, for instance, prevalent in bipolar patients [42]. The negative impact of poor sleep on mood and cognitive functioning is particularly noticeable in bipolar patients [43]. Fitbit smartwatches collect sleep duration and stages, which can help us in measuring the impact of sleep quality on next-day cognitive performance. Literature suggests a relationship between reduced HRV and impairment in inhibition control [44]. Moreover, reduced HRV was observed in bipolar and schizophrenic patients compared with healthy controls [45]. Our outlook is to create a *Cognitive Watch* to extend human knowledge regarding cognition.

### Limitations

The limitations of this study are threefold. First, this study was conducted with 21 healthy adults who were recruited mostly from the campus of the Technical University of Denmark. This was deemed appropriate for evaluating the UbiCAT as compared to existing tools. However, studies involving patients and people with cognitive impairment are needed and are the focus of our upcoming studies. Second, the UbiCAT is designed for in-the-wild administration and, yet, this study was conducted in an indoor environment. This was done because cognitive performance fluctuates and in order to be able to assess cognition using both UbiCAT and the computer-based tests, the tests had to be administered right after each other on both platforms in order to achieve comparable measures. Therefore, moving the participants inside and outside between computer-based and smartwatch-based test sessions could yield unreliable cognitive measures for our study. In our upcoming studies, however, the UbiCAT will be used outside the clinic in order to collect real-world cognitive performance, which will be compared with cognitive assessments performed in a clinic. Third, the results indicated that the UbiCAT tests may not reflect the optimal performance of the participants compared to the computer-based tests. Nevertheless, frequent tests with the UbiCAT in upcoming studies and with various patient groups may better verify the optimal performance of the UbiCAT users.

### Conclusions

In this study, the UbiCAT as a smartwatch-based tool for cognitive assessment was evaluated against computer-based cognitive assessment tools. The results revealed significant correlations between the total scores of the UbiCAT tests and standard computer-based tests. The psychometric factors regarding the aesthetics, functionality, and information quality and quantity of the apps yielded high usability ratings from the study participants. The majority of our study participants felt comfortable when using the UbiCAT. The findings of this study showed that the UbiCAT can be used for assessing attention, working memory, and executive function across participants' everyday lives, along with mobile data collection. Future studies can administer the UbiCAT to mentally ill patients to collect their daily cognitive functioning data and to compare their results with lab-based studies.

## Appendix

Multimedia Appendix 1 Usability evaluation questionnaire.

Multimedia Appendix 2 Analysis of variance on the N-back test results.

Multimedia Appendix 3 Themes and their corresponding quotes extracted from the interviews.

## Abbreviations

ADHD: attention deficit hyperactivity disorder
ANOVA: analysis of variance
API: application programming interface
BACS: Brief Assessment of Cognition in Schizophrenia
CANTAB: Cambridge Neuropsychological Test Automated Battery
CST: Color-Shape Test
EDTB: Edinburgh Delirium Test Box
EMA: ecological momentary assessment
ESM: experience-sampling method
HRV: heart rate variability
ICAT: Internet-based Cognitive Assessment Tool
MARS: Mobile App Rating Scale
MyCQ: MyCognition Quotient
NASA TLX: NASA Task Load Index
OS: operating system
PVT: psychomotor vigilance task
PVT-B: brief version of the psychomotor vigilance task
RT: response time
THINC-it: THINC-integrated tool
UbiCAT: Ubiquitous Cognitive Assessment Tool
UDS: Uniform Data Set
