# Supplementary material for: The Ubiquitous Cognitive Assessment Tool for Smartwatches: Design, Implementation, and Evaluation Study
Source: JMIR Mhealth Uhealth. 2020 Jun 1;8(6):e17506. doi: 10.2196/17506 (PMC7296405; doi:10.2196/17506)
Supplement: Multimedia Appendix 1 [file mhealth_v8i6e17506_app1.pdf]

Usability Evaluation

* Required

Aesthetics

1. **Is arrangement and size of buttons/icons/content on the screen appropriate?** *
   Mark only one oval.

   - Very bad design, cluttered, some options impossible to select/locate/see/read device display not optimised
   - Bad design, random, unclear, some options difficult to select/locate/see/read
   - Satisfactory, few problems with selecting/locating/seeing/reading items or with minor screen-size problems
   - Mostly clear, able to select/locate/see/read items
   - Professional, simple, clear, orderly, logically organised, device display optimised. Every design component has a purpose

2. **How high is the quality/resolution of graphics used for buttons/icons/content?** *
   Mark only one oval.

   - Graphics appear amateur, very poor visual design - disproportionate, completely stylistically inconsistent
   - Low quality/low resolution graphics; low quality visual design – disproportionate, stylistically inconsistent
   - Moderate quality graphics and visual design (generally consistent in style)
   - High quality/resolution graphics and visual design – mostly proportionate, stylistically consistent
   - Very high quality/resolution graphics and visual design - proportionate, stylistically consistent throughout

3. **How good does the app look?** *
   Mark only one oval.

   - No visual appeal, unpleasant to look at, poorly designed, clashing/mismatched colours
   - Little visual appeal – poorly designed, bad use of colour, visually boring
   - Some visual appeal – average, neither pleasant, nor unpleasant
   - High level of visual appeal – seamless graphics – consistent and professionally designed
   - As above + very attractive, memorable, stands out; use of colour enhances app features/menus

Functionality

4. **Performance: How accurately/fast do the app features (functions) and components (buttons/menus) work?** *
Mark only one oval.

- ( ) App is broken; no/insufficient/inaccurate response (e.g. crashes/bugs/broken features, etc.)
- ( ) Some functions work, but lagging or contains major technical problems
- ( ) App works overall. Some technical problems need fixing/Slow at times
- ( ) Mostly functional with minor/negligible problems
- ( ) Perfect/timely response; no technical bugs found/contains a 'loading time left' indicator

5. **Ease of use: How easy is it to learn how to use the app; how clear are the menu labels/icons and instructions?** *
Mark only one oval.

- ( ) No/limited instructions; menu labels/icons are confusing; complicated
- ( ) Useable after a lot of time/effort
- ( ) Useable after some time/effort
- ( ) Easy to learn how to use the app (or has clear instructions)
- ( ) Able to use app immediately; intuitive; simple

Information

6. **Quality of information: Are instructions content correct, well written, and relevant to the goal/topic of the app?** *
Mark only one oval.

- ( ) Irrelevant/inappropriate/incoherent/incorrect
- ( ) Poor. Barely relevant/appropriate/coherent/may be incorrect
- ( ) Moderately relevant/appropriate/coherent/and appears correct
- ( ) Relevant/appropriate/coherent/correct
- ( ) Highly relevant, appropriate, coherent, and correct

7. **Quantity of information: Is the extent coverage within the scope of the app and comprehensive but concise?** *
Mark only one oval.

- ( ) Minimal or overwhelming
- ( ) Insufficient or possibly overwhelming
- ( ) OK but not comprehensive or concise
- ( ) Offers a broad range of information, has some gaps or unnecessary detail; or has no links to more information and resources
- ( ) Comprehensive and concise; contains links to more information and resources

Powered by
