# Supplementary material for: The Ubiquitous Cognitive Assessment Tool for Smartwatches: Design, Implementation, and Evaluation Study
Source: JMIR Mhealth Uhealth. 2020 Jun 1;8(6):e17506. doi: 10.2196/17506 (PMC7296405; doi:10.2196/17506)
Supplement: Multimedia Appendix 2 [file mhealth_v8i6e17506_app2.pdf]

Analysis of Variance on the N-Back Test Results

One-way ANOVA was performed to analyze the effect of difficulty level on the participants' test performance. The analysis on the number of correct responses of the participants in the Letter test revealed a significant effect of the difficulty level ($F(2,60)=32.20$, $p<0.001$). Post-hoc comparisons using the Bonferroni correction test revealed that participants received higher scores in 1-back compared to 2-back (Mean Difference (MD)= 5.71, SE= 1.16, $p<.001$, Cohens's d= 1.38) and 3-back (MD=9.19, SE=1.16, $p<0.001$, d= 2.34). The analysis showed that the number of correct responses of 2-back were higher than 3-back (MD=3.48, SE= 1.16, p=0.012, d= 1.13). The corresponding outcomes for the Psytoolkit N-back tests showed that the effect of difficulty level on the number of correct responses of the participants was also significant ($F(2,60)=20.93$, $p<0.001$). No statistically significant difference was found between the number of correct responses in 1-back and 2-back (p=0.57) while the participants were less accurate in 3-back compared to the 1-back (MD=6.86, SE=1.12, $p<0.001$, d= 1.66) and 2-back (MD=5.38, SE=1.12, $p<0.001$, d= 1.96).

The analysis of variance showed that the effect of difficulty level on the mean RTs of the participants in the Letter test was also significant ($F(2,60)=13.79$, $p<0.001$). Post-hoc comparisons using the Bonferroni correction test showed that participants performed faster in 1-back comparing to 2-back (MD=-352.19, SE=67.89,$p<0.001$, d= -1.48) and 3-back (MD=-224.24, SE=67.89,p=0.005, d= -0.99). We did not find a significant difference between the mean RTs of the 2-back and 3-back (p=0.20). The corresponding analysis for the Psytoolkit N-back tests showed that the effect of difficulty level on the mean RTs of the participants was also significant($F(2,60)=15.33$, $p<0.001$). Participants responded faster in 1-back comparing to 2-back (MD=-314.48, SE=64.31, $p<0.001$, d= -1.60) and 3-back (MD=-301.95, SE=67.89, $p<0.001$, d= -1.48) while their mean RTs in the 2-back and 3-back were not statistically different (p=1.00).
