# Supplementary material for: The Ubiquitous Cognitive Assessment Tool for Smartwatches: Design, Implementation, and Evaluation Study
Source: JMIR Mhealth Uhealth. 2020 Jun 1;8(6):e17506. doi: 10.2196/17506 (PMC7296405; doi:10.2196/17506)
Supplement: Multimedia Appendix 3 [file mhealth_v8i6e17506_app3.pdf]

Table 1A: The themes extracted from the interviews as well as participants' quotes related to each theme.

Theme	Quotes
**Perception about the Experiment**	P19:“I think it should be every other day to test different things.”
**Input Modality**	P1:“The Color test is better on the watch because although I know where the letters are on the keyboard, they still should have used some other thing. It makes sense to input it [the color] as buttons, not keyboard.”<br><br>P9:“The first task, Arrow test, was more comfortable on the watch. Even though I knew where keys located, I realized that I looked down quite often just to check if I am pressing the correct key. The distance is more with the computer. Also for the Letter test, it was more comfortable on the watch. But for the last task (Color test) I felt more comfortable on the keyboard because I noticed on the watch I missed a button couple of times. But in computer I had plenty of keys to press on the keyboard, at the same time sacrificed some time to find the correct key. Overall, I would say that touch screen makes a huge difference and it is better to use a touch screen.”
**Device Screen**	P14: “I think the big screen helped me but I felt alright to use the smartwatch. I like that the screen was small, simplified [to] what you need to do.”<br>P16: It is much easier with the computer because it is bigger and I am more used to [it].”
**Visual Impact**	P15: “I think the computer is better for me because you can see more clearly. The most uncomfortable thing is my eyes, not my hand. Staring at a small screen is not comfortable. The N-back [test] is much easier on the computer and my brain can store it better. ”<br>P21: “I am to be honest not a fan of the Fitbit visual. Comparing to [Anonymous smartwatch company], the colors and the fonts are not that appealing. It is the Fitbit's problem as I see.“

**Psychological Factors**	P4: “The smartwatch is nice for short time use. One-back was easier on the watch, I think. When I lost [a score] for 2- and 3-back, then I had more wrong answers on the smartwatch as compared to the computer. I found it more playful on the watch, more like a game compared to the computer. [On the] computer, it did not feel like playing - not as emotional.“
**Performance**	P3: “I could not clearly understand the instructions [of the Letter test] and I made mistakes. Also maybe because I am a novice user of the watch, I sometimes felt that tapping was not accurate.”<br>P14: “I think the first results are worse than the last results. It takes a little bit of time to get accustomed.”
**Suggestions**	P21: “The last test [Color test] font size was so narrow. Making the color names bold is better.”
